# A novel prognostic 6-gene signature for osteoporosis

**DOI:** 10.3389/fendo.2022.968397

**Published:** 2022-09-21

**Authors:** Yu Zhao, Jieping Yan, Yimiao Zhu, Zhenping Han, Tingting Li, Lijuan Wang

**Affiliations:** ^1^ Geriatric Medicine Center, Department of Endocrinology, Zhejiang Provincial People’s Hospital (Affiliated People’s Hospital, Hangzhou Medical College), Hangzhou, Zhejiang, China; ^2^ Key Laboratory of Endocrine Gland Diseases of Zhejiang Province, Hangzhou, Zhejiang, China; ^3^ Center for Clinical Pharmacy, Cancer Center, Department of Pharmacy, Zhejiang Provincial People’s Hospital (Affiliated People’s Hospital, Hangzhou Medical College), Hangzhou, Zhejiang, China; ^4^ Center for General Practice Medicine, Department of Gastroenterology, Zhejiang Provincial People’s Hospital (Affiliated People’s Hospital, Hangzhou Medical College), Hangzhou, Zhejiang, China; ^5^ Department of Pharmacology, College of Pharmaceutical Sciences, Zhejiang University of Technology, Hangzhou, Zhejiang, China; ^6^ Center for General Practice Medicine, Department of Rheumatology and Immunology, Zhejiang Provincial People’s Hospital (Affiliated People’s Hospital, Hangzhou Medical College), Hangzhou, Zhejiang, China

**Keywords:** osteoporosis, GEO datasets, machine learning, diagnosis, WGCNA

## Abstract

**Introduction:**

The incidence of osteoporosis (OP) keeps increasing due to global aging of the population. Therefore, identifying the diagnostic and prognostic biomarkers of OP is of great significance.

**Methods:**

mRNA data from OP and non-OP samples were obtained from GEO database, which were divided into training set (GSE35959) and testing sets (GSE7158, GSE62402, GSE7429 and GSE56815). Gene modules most significantly related to OP were revealed using weighted gene co-expression network analysis (WGCNA) and differentially expressed genes (DEGs) between OP and normal samples in training set were identified using limma R package. Thereafter, above two gene sets were intersected to obtain the genes potentially related to OP. Protein-protein interaction (PPI) pairs were screened by STRING database and visualized using Cytoscape, while the plug-in cytoHubba was used to screen hub genes by determining their topological parameters. Afterwards, a diagnostic model was constructed using those hub genes, whose creditability was further evaluated by testing sets.

**Results:**

The results of WGCNA analysis found the Black module was most significantly related to OP, which included altogether 1286 genes. Meanwhile, 2771 DEGs were discovered between OP patients and the normal controls. After taking the intersection, 479 genes were identified potentially correlated with the development of OP. Subsequently, six hub genes were discovered through PPI network construction and node topological analysis. Finally, we constructed a support vector machine model based on these six genes, which can accurately classified training and testing set samples into OP and normal groups.

**Conclusion:**

Our current study constructed a six hub genes-based diagnostic model for OP. Our findings may shed some light on the research of the early diagnosis for OP and had certain practical significance.

## Introduction

Osteoporosis (OP) is a systemic skeletal disorder characterized by the destructed microstructure of bone tissue, reduced bone mass, higher risk of fracture, and elevated bone fragility (1). As reported by the National Health and Nutrition Examination Survey III, the U.S. has witnesses an annual of over 9.9 million OP cases, including 1.5 million with osteoporotic fractures (2). With the aging of population, it is expected that the OP-related social costs will increase (3). OP has a hidden onset and will cause disability (4). No symptom is observed in many cases at the early-middle stages. But acute osteoporotic fracture will result in lifetime disability (5). Detecting and treating OP at the early stage remarkably improve the patients’ survival and life quality (6). Nonetheless, little research is conducted to explore the pathogenic mechanism of OP. Numerous factors, like changed estrogen signaling (7) and oxidative stress (8), are suggested to induce OP, but the early diagnostic and therapeutic biomarkers for OP are not discovered by now. Consequently, it is urgently needed to identify and develop diagnostic and therapeutic biomarkers for OP with high sensitivity and specificity.

In recent years, many individual OP-associated genes are recognized as the candidate biomarkers for diagnosis (9, 10). Most studies aim to screen differentially expressed genes (DEGs) as biomarkers (11–13). Model analysis, which is frequently applied when there are additional covariates, is extensively adopted for improving prediction probability (14). Up to now, genes with similar regulatory function in OP have not been widely explored. Weighted gene co-expression network analysis (WGCNA) is a latest systems biology approach with particular practicability under such circumstances, which can assist in establishing the free-scale gene co-expression networks for identifying the relationships of gene sets with clinical features or among diverse gene sets (15, 16). WGCNA has been widely adopted to identify clinical features-associated hub genes for diverse disorders, such as heart failure (17), gastric cancer (18) and ovarian cancer (19).

**Figure 1:**
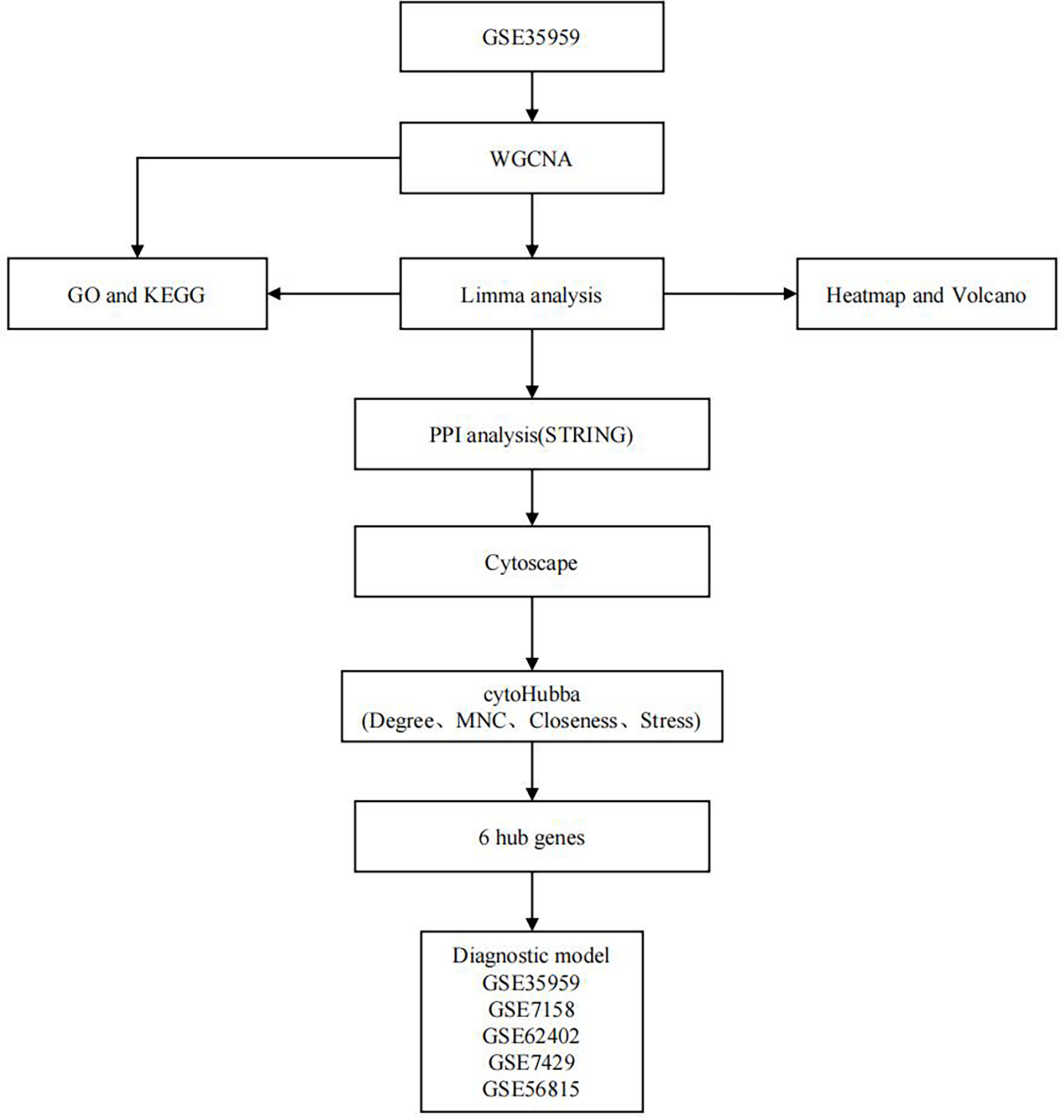
Flowchart of this study.

This work constructed a creditable diagnostic model by using the identified hub genes (based on WGCNA, DEG analysis, PPI network construction and topological analysis; **Figure 1**). This model was significantly related to OP development and can effectively distinguish OP cases from normal ones. Our findings may shed some light on the research of the early diagnosis for OP and have certain practical significance.

## Materials and methods

### Data extraction

RNA expression data were obtained from Gene Expression Omnibus (GEO, https://www.ncbi.nlm.nih.gov/geo/) database (accession number: GSE35959, GSE7158, GSE7429, GSE62402 and GSE56815). The details are as follows (1): GSE35959 contained 14 Normal samples and five OP samples (2); GSE7158 contained 14 high peak bone mass samples and 12 low peak bone mass samples (3); GSE62402 contained five high bone mineral density (BMD) samples and five low BMD samples (4); GSE7429 contained 10 high BMD samples and 10 low BMD samples (5); GSE56815 covered 40 high BMD samples and 40 low BMD samples.

### Weighted gene co-expression network analysis

WGCNA was conducted using the free R package (20). We determined the Pearson coefficients between two arbitrary genes. Then, multiple powers of N were selected for correlation coefficients to align inter-gene connections with scale-free network distribution. Moreover, we established the network by one-step function and detected the consensus modules. The power β = 5 (scale-free = 0.94) was selected as the soft threshold to ensure the scale-free network. Further, the inter-gene correlation coefficients were utilized to construct a hierarchical clustering tree. The diverse branches in the clustering tree stood for diverse gene modules, while modules were distinguished by the diverse colors. Subsequently, this study determined Module Eigengene (ME) values for all modules, together with correlation coefficient between dichotomous phenotypes (normal or OP) and ME. P<0.05 stood for statistical significance in correlation analysis between phenotype and ME. A higher ME absolute value indicated its tighter correlation with the specific phenotype.

### Discovery and verification of DEGs

This work adopted the random variance model t-test, which efficiently elevated the small sample degree of freedom (DOF), for filtering DEGs between normal and OP samples in training set (21). Thereafter, DEGs were chosen following false discovery rate and significance analyses based on the thresholds of |FC| >1.5 and p<0.05. Heatmaps were plotted to visualize results.

### Functional annotation and pathway enrichment analyses

The R language WebGestalt R function package (22) was adopted to conduct Gene Ontology (GO) terms (including biological process, cellular component, molecular function) as well as Kyoto Encyclopedia of Genes and Genomes (KEGG) analyses (23–25) on DEGs between normal and OP samples and genes related to critical module. P<0.05 stood for statistical significance.

### PPI network establishment

STRING (version 11.0, https://string-db.org/) (26) has been developed as the extensively utilized database for analyzing and predicting protein interactions and functional connections in the context of human disorder. We adopted STRING to analyze the protein interactions and functional connections through DEGs analysis and WGCNA, and selected those interaction pairs whose confidence scores were ≥ 0.4. Then, Cytoscape software (https://cytoscape.org/index.html, version 3.7.2) was adopted to visualize PPI network according to previous description, while cytoHubba plug-in (27) was employed to screen hub genes within the PPI network based on the node topological parameters. Nodes with top 20 degree, MNC, Closeness and Stress values among all nodes in the network were deemed as the hub nodes in the network; in other words, they were the hub genes most significantly related to OP in this study.

### Statistical analysis

SPSS23.0 (SPSS, Chicago, IL, USA) was utilized for data analysis. Wilcoxon tests were utilized to compare statistical differences of two groups, whereas abnormally distributed variables were compared by unpaired Student t-tests. Pearson and distance correlation analysis was adopted to determine correlation coefficients. Due to the small sample size, following multiple-testing controls, excessively large adjusted p-values were obtained. The raw p<0.05 was utilized to be the threshold to select DEGs. This study selected false discovery rate <0.05 after multiple testing adjustment for filtering those related GO terms as well as KEGG pathways.

## Results

### The black module was tightly associated with OP incidence in training set upon WGCNA

**Figure 2:**
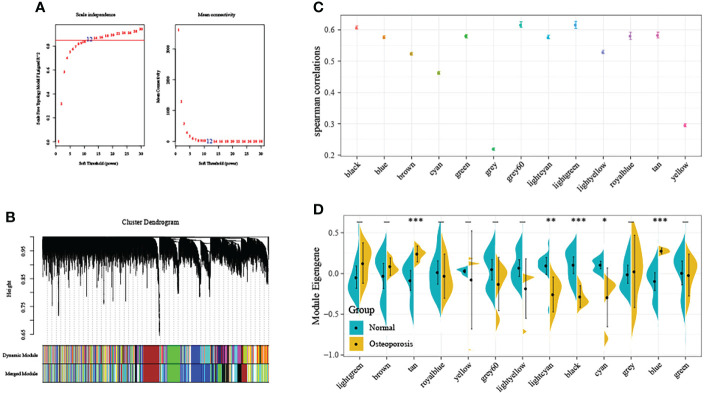
Weighted gene co-expression network analysis (WGCNA). **(A)** Selection of the soft threshold in the WGCNA. The red line represents the correlation coefficient, and the first point above the red line is the soft threshold (β = 5). **(B)** Clustering of the gene modules. Each designated color represents a gene module, and the genes that could not be grouped into other modules were placed in the grey module. **(C)** The correlation between ME gene modules and dichotomous phenotypes. **(D)** The distribution difference of ME in each module between disease and healthy samples. *P<0.05, **P<0.01, ***P<0.001.

First of all, this study normalized GSE35959 expression data in training set for removing those batch effects, and there was no distinct deviation detected across the diverse samples after normalization. As a result, we utilized the normalized data in later analyses. The co-expression network has been previously suggested to be consistent with the unsigned network characteristics. Consequently, this study set the soft threshold at 5 for ensuring the unsigned co-expression network (**Figure 2A**). According to the mixed dynamic shear tree criteria, we set the gene module minimum cardinality at 50. Then, we utilized an average-linkage hierarchical clustering approach for clustering genes and determined ME values of all modules. Thereafter, we performed module clustering analysis, and combined close modules to obtain a novel module whose setting height was 0.25. We acquired altogether 13 modules, among which, genes in the gray module were not divided to any module (**Figure 2B**). Subsequently, we used two calculation methods to determine the correlation between OP and the module. First, we calculated the Spearman correlation coefficient between the ME of each module and OP, and modules with the median correlation coefficients >0.6 were selected (**Figure 2C**). Second, we analyzed the distribution difference of ME in each module between disease and healthy samples. As observed, the distributions of feature vectors in OP samples of tan and blue modules were significantly higher than those in healthy control, whereas those of lightcyan, black and cyan modules were markedly lower than those in healthy group (**Figure 2D**). Typically, the most significant difference in distribution was observed in black module between two groups of samples. According to the above findings, genes (n=1286) in black module might be potentially associated with OP occurrence.

**Figure 3:**
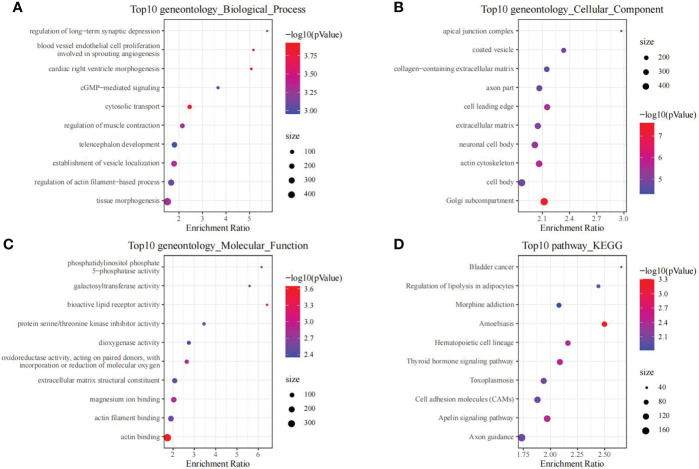
The functional analysis of genes in black module. GO biological process **(A)**, GO cellular component **(B)**, GO molecular function **(C)** and KEGG **(D)** enrichment analysis of genes in the black module.

Moreover, we also conducted enrichment analysis on genes in black module, so as to discover the related regulatory biological functions and signal pathways. According to our results, the 1286 genes were enriched into 69 biological processes (including telencephalon development, regulation of muscle contraction, and cGMP-mediated signaling), 26 molecular functions, 127 cellular components and 11 signal transduction pathways (such as Cell adhesion molecules, Apelin signaling pathway, and Thyroid hormone signaling pathway). The top 10 GO and KEGG terms with the highest significance are presented in **Figure 3**.

### DEGs in training set

**Figure 4:**
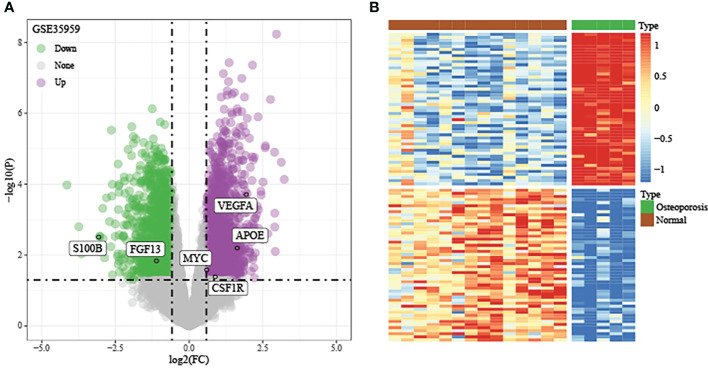
Discovery and verification of DEGs between OP samples and normal samples in training set. **(A)** Volcano plot of significantly DEGs. **(B)** Heat map of DEGs.

**Figure 5:**
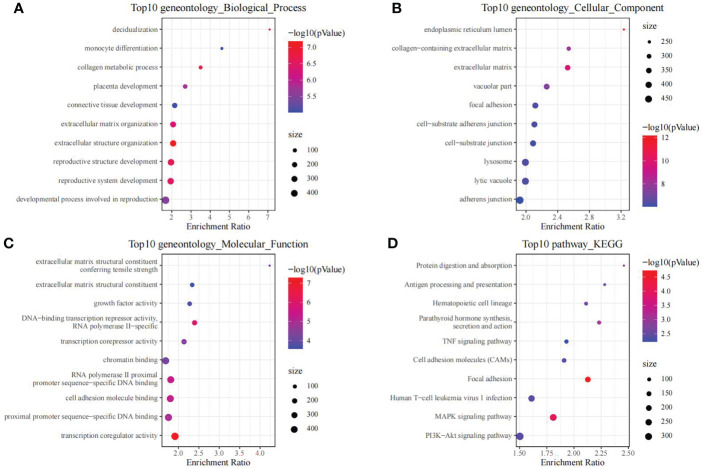
The functional analysis of upregulated DEGs between OP samples and normal samples in training set. GO biological process **(A)**, GO cellular component **(B)**, GO molecular function **(C)** and KEGG **(D)** enrichment analysis of DEGs.

**Figure 6:**
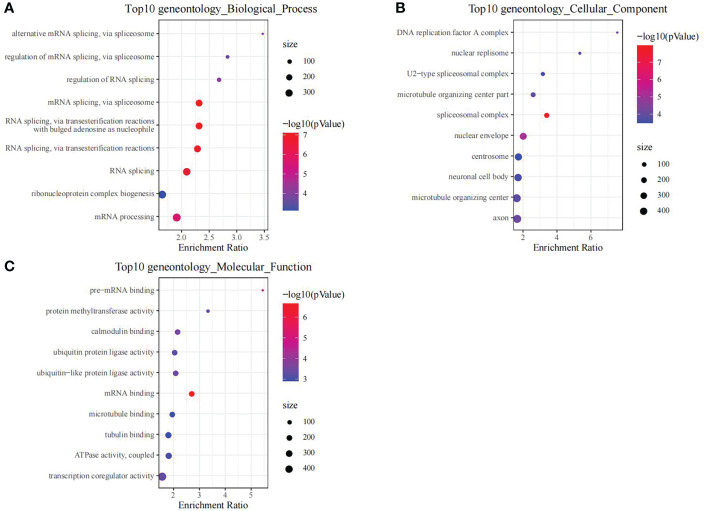
The functional analysis of downregulated DEGs between OP samples and normal samples in training set. GO biological process **(A)**, GO cellular component **(B)** and GO molecular function **(C)** enrichment analysis of DEGs.

Using the thresholds of p<0.05 and |FC|>1.5, we discovered 2771 DEGs between normal and OP samples (**Figure 4**). As revealed by pathway analysis, those DEGs were mostly related to the Focal adhesion and TNF/MAPK signaling pathways. According to GO functional annotation, those up-regulated DEGs were related to extracellular structure organization and collagen metabolism. In addition, those down-regulated DEGs were related to ribonucleoprotein complex biogenesis and mRNA splicing (As shown in **Figure 5** and **Figure 6**).

### OP-associated hub gene selection from training set

**Figure 7:**
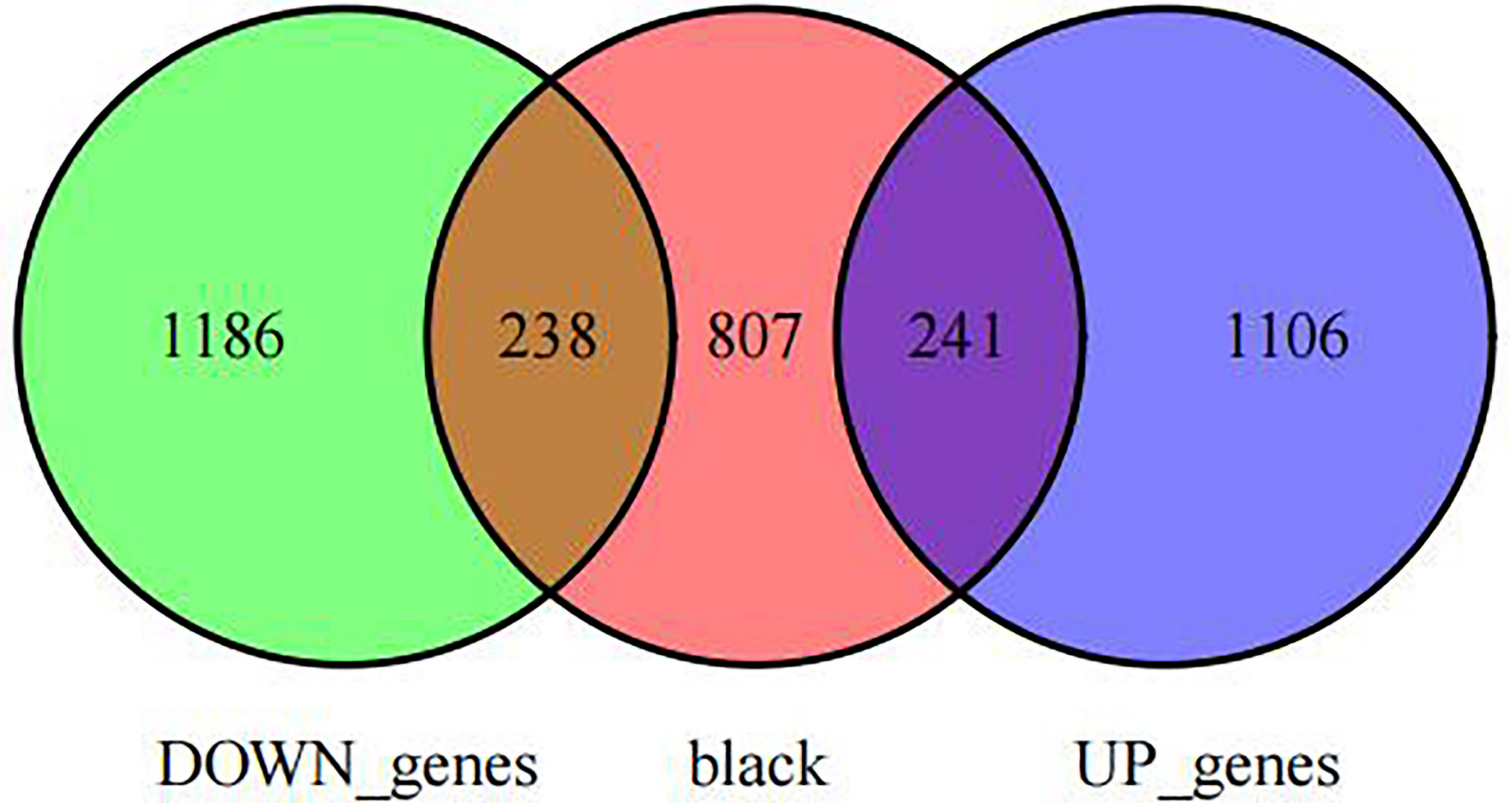
Venn diagram of intersections between co-expressed genes and DEGs.

**Figure 8:**
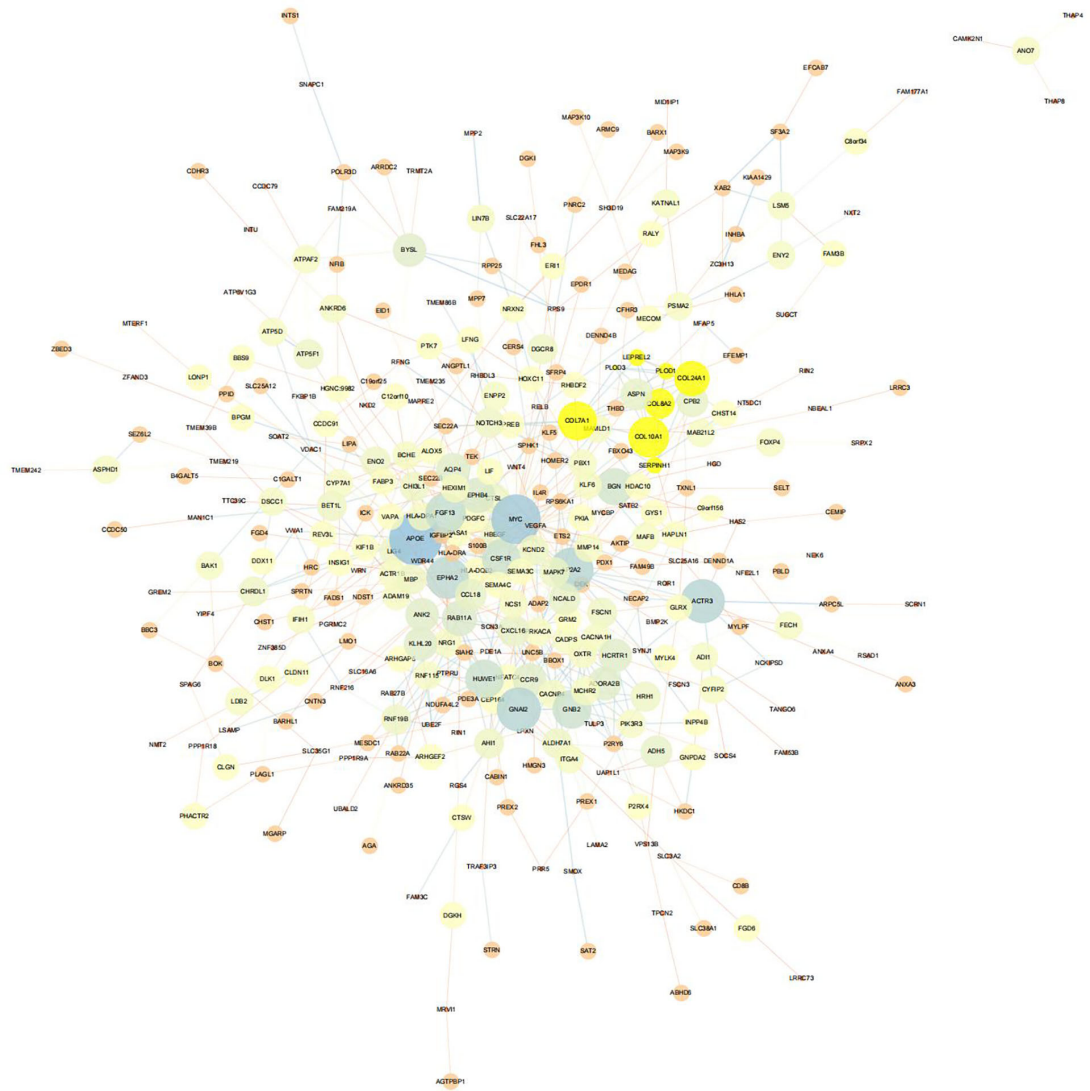
PPI network of 479 genes potentially related to OP.

**Figure 9:**
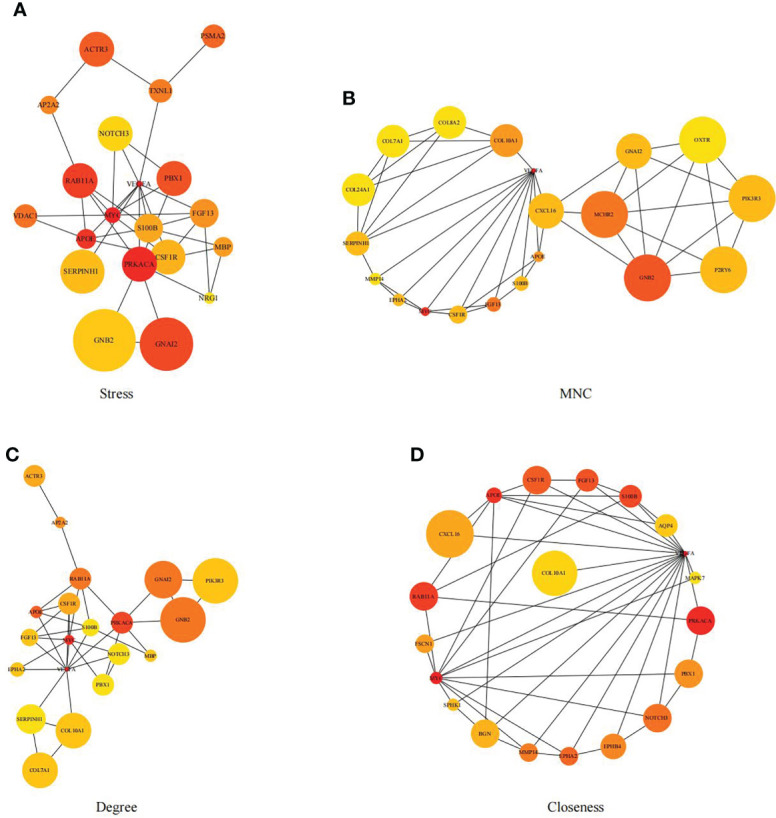
Genes with top 20 stress **(A)**, MNC **(B)**, degree **(C)** and closeness **(D)** values were shown.

**Figure 10:**
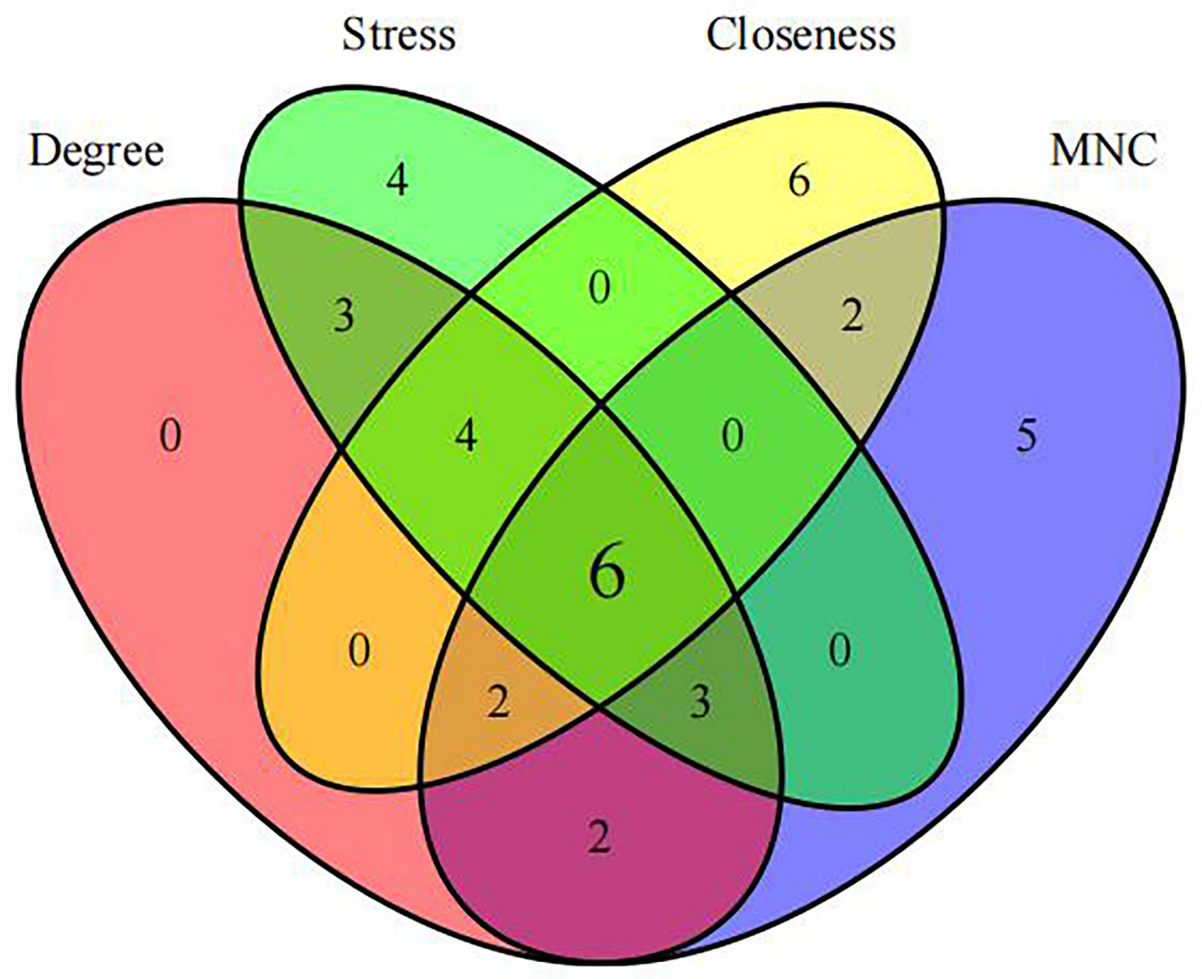
Identification of hub genes.

First, we intersected DEGs with gene set in the black module to obtain 479 potential OP-related genes (**Figure 7**). Subsequently, for identifying OP-related hub genes, we mapped altogether 479 genes to String database, those with the confidence scores ≥ 0.4 were screened. Typically, the nodes in the network stood for individual genes, whereas the edges stood for gene-gene interactions. Later, Cytoscape was employed for visualization (**Figure 8**). Thereafter, we adopted the cytoHubba plug-in for analyzing the node topological properties, and genes with top 20 degree, MNC, closeness and stress values were screened (**Figure 9** and **Figure 10**, n=6, including MYC, VEGFA, CSF1R, S100B, APOE and FGF13) to be the hub genes related to OP genesis and progression.

### Performance of the diagnostic model in distinguishing normal samples and OP samples

**Figure 11:**
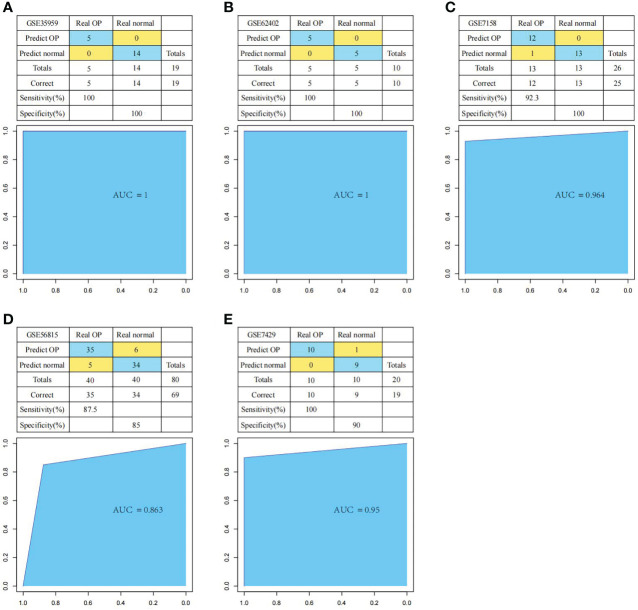
Performance of the diagnostic model in distinguishing normal samples and OP samples. The classification results and the ROC curves of the diagnostic model for set GSE35959 **(A)**, GSE62402 **(B)**, GSE7158 **(C)**, GSE56815 **(D)** and GSE7429 **(E)**.

First of all, we constructed the OP diagnostic model based on the SVM classification model according to the expression profiles of these six genes. We applied the training set samples for verification. The results suggested that the model classification accuracy rate was 100%, all the 19 samples were correctly classified, with the model sensitivity, specificity, and area under the ROC curve (AUC) of 100%, 100% and 1, respectively (**Figure 11A**). Thereafter, we used multiple test set samples to verify the model classification accuracy and sensitivity. The 10 samples in test set GSE62402 were correctly classified, with the accuracy rate, sensitivity, specificity, and AUC of 100%, 100%, 100% and 1, respectively (**Figure 11B**). Among the 26 samples in test set GSE7158, 25 were correctly classified, with the accuracy rate, sensitivity, specificity and AUC of 96.2%, 92.3%, 100% and 0.964, respectively (**Figure 11C**). For the 80 samples in test set GSE56815, 69 were correctly classified, with the accuracy rate, sensitivity, specificity and AUC of 86.3%, 87.5%, 85% and 0.863, separately (**Figure 11D**). For the 20 samples in test set GSE7429, 19 were correctly classified, with the accuracy rate, sensitivity, specificity, and AUC of 95%, 100%, 90% and 0.95, respectively (**Figure 11E**). These results suggested that the diagnosis prediction model constructed based on six hub genes effectively distinguished OP samples from normal samples, and these six genes might serve as the reliable biomarkers for OP diagnosis.

## Discussion

OP accounts for a frequently seen and complicated systemic bone disorder, which is more usually seen in female population (28). OP has a hidden onset, which is usually detected in the late stages. In the case of secondary osteoporotic fracture, numerous complications will develop, and patients are associated with dismal prognostic outcome. Consequently, numerous investigators explore the OP diagnosis at molecular level, therapeutic targets and genetic modulation. The present work discovered 479 OP-related genes by differential expression analysis and WGCNA. At the same time, we also conducted functional annotation, which revealed that such genes were mostly enriched into biological processes (like body immunity, cell adhesion, and extracellular matrix remodeling) and related signaling pathways. The immune and bone systems have been suggested to function as the close-knit functional unit (osteoimmune system) because of the shared developmental niche. Dysregulated immunity is related to the occurrence of diverse inflammatory autoimmune disorders that adversely affect bone integrity like OP (29). Besides, T cells have been found to be related to pathological bone loss associated with diverse disorders like rheumatoid arthritis, bisphosphonate-related jaw osteonecrosis and ovariectomy-induced bone loss (30). Moreover, multiple factors in extracellular matrix (ECM, including osteopontin and calnexin) have been proved to regulate osteoclast and osteoblast functions to achieve chemotaxis of multiple immune cells, thus participating in OP genesis and development (31).

Thereafter, this study discovered six hub genes associated with OP progression based on topological features from the as-constructed PPI network. Among them, four genes (*VEGF*, *MYC*, *CSF1R* and *APOE*) were closely related to and participated in OP development. *VEGF* has been verified to promote angiogenesis in local focus site, accelerate bone formation and reconstruction, and directly promote the differentiation of bone marrow mesenchymal stem cells (BMSCs) increase the osteogenic activity of osteoblasts to promote bone formation and increase bone density (32, 33). *MYC* is discovered to regulate the BMSC proliferation and osteogenic differentiation (34). The high expression of *CSF1R* and its ligand *CSF1* is verified to promote the macrophage-mediated inflammatory response, aggravate bone loss and thus deteriorate OP (35). Apolipoprotein E (APOE) genotypes are found to be related to OP. The susceptibility haplotype *CGT* is suggested to be tightly related to BMD as well as the incidence of OP and osteopenia among postmenopausal women (36, 37). The above findings suggest that the four hub genes were possibly related to human disorder genesis and progression, like OP. Chromosomal rearrangement and expression changes of *S100B* gene have been verified to be related to several nervous system diseases, neoplastic diseases, endocrine diseases like Alzheimer’s disease (AD), Down’s syndrome, epilepsy, amyotrophic lateralizing sclerosis, tumor and type 1 diabetes (38, 39). In addition, *FGF13* has extensive activities in promoting mitosis and cell survival, which participates in numerous biological processes, including embryonic development, cell growth, morphogenesis, tissue repair, tumor growth and invasion (40). But there is no research validating the functions of the two genes in OP at present. At last, this work constructed a diagnostic model by incorporating six genes, which effectively distinguished normal samples from OP samples.

Studies with larger sample sizes are warranted for evaluating our model reliability. Besides, the functions of *FGF13* and *S100B* in OP development must be further examined in future studies.

## Conclusion

To sum up, this study constructed a creditable diagnostic model by incorporating six critical genes like *MYC*, *VEGFA*, *CSF1R*, *S100B*, *APOE* and *FGF13*, which effectively distinguished normal samples and OP samples. According to this study, our constructed diagnostic model can be used to diagnose OP in clinic.

## Data availability statement

The datasets presented in this study can be found in online repositories. The names of the repository/repositories and accession number(s) can be found below: https://www.ncbi.nlm.nih.gov/geo/, GSE35959, GSE7158, GSE7429, GSE62402 and GSE56815.

## Author contributions

LW conceived and designed the study, YZ and JY analyzed the data and prepared the manuscript, YMZ and ZH collected the data and organized the figures, and TL edited the manuscript. All authors contributed to the article and approved the submitted version.

## Funding

This study was supported by National Natural Science Foundation for Youth of China (81903597), Zhejiang Provincial Natural Science Foundation for Youth of China (LQ16H310003), Medical and Health Science and Technology Research Program of Zhejiang Province (2019RC096, 2021KY018, 2021KY016), and Zhejiang Provincial Department of Education General Project (Y202044708).

## Conflict of interest

The authors declare that the research was conducted in the absence of any commercial or financial relationships that could be construed as a potential conflict of interest.

## Publisher’s note

All claims expressed in this article are solely those of the authors and do not necessarily represent those of their affiliated organizations, or those of the publisher, the editors and the reviewers. Any product that may be evaluated in this article, or claim that may be made by its manufacturer, is not guaranteed or endorsed by the publisher.
